# Loss of Endometrial Sodium Glucose Cotransporter SGLT1 is Detrimental to Embryo Survival and Fetal Growth in Pregnancy

**DOI:** 10.1038/s41598-017-11674-3

**Published:** 2017-10-03

**Authors:** Madhuri S. Salker, Yogesh Singh, Ni Zeng, Hong Chen, Shaqiu Zhang, Anja T Umbach, Hajar Fakhri, Ursula Kohlhofer, Leticia Quintanilla-Martinez, Ruban R. Peter Durairaj, Flavio S. V. Barros, Pavle Vrljicak, Sascha Ott, Sara Y Brucker, Diethelm Wallwiener, Ivana Vrhovac Madunić, Davorka Breljak, Ivan Sabolić, Hermann Koepsell, Jan J. Brosens, Florian Lang

**Affiliations:** 1 Department of Physiology I, Eberhard-Karls Universität Tübingen, Tübingen, D-72076 Germany; 2 Obstetrics, Gynecology, Eberhard-Karls Universität Tübingen, Tübingen, D-72076 Germany; 3 Institute of Pathology and Comprehensive Cancer Centre, Eberhard-Karls Universität Tübingen, Tübingen, D-72076 Germany; 4 Division of Biomedical Sciences, Warwick Medical School, and Tommy’s National Centre for Miscarriage Research, University Hospitals Coventry and Warwickshire NHS Trust, Clifford Bridge Rd, Coventry, CV2 2DX UK; 5 Warwick Systems Biology Centre, University of Warwick, Coventry, CV4 7AL UK; 6 Molecular Toxicology Unit, Institute for Medical Research and Occupational Health, 10000 Zagreb, Croatia; 7 Department of Molecular Plant Physiology and Biophysics, Julius-von-Sachs-Institute, University of Würzburg, Würzburg, D-97082 Germany

## Abstract

Embryo implantation requires a hospitable uterine environment. A key metabolic change that occurs during the peri-implantation period, and throughout early pregnancy, is the rise in endometrial glycogen content. Glycogen accumulation requires prior cellular uptake of glucose. Here we show that both human and murine endometrial epithelial cells express the high affinity Na^+^-coupled glucose carrier SGLT1. Ussing chamber experiments revealed electrogenic glucose transport across the endometrium in wild type (*Slc5a1* ^+/+^) but not in SGLT1 deficient (*Slc5a1* ^−/−^) mice. Endometrial glycogen content, litter size and weight of offspring at birth were significantly lower in *Slc5a1* ^−/−^ mice. In humans, *SLC5A1* expression was upregulated upon decidualization of primary endometrial stromal cells. Endometrial *SLC5A1* expression during the implantation window was attenuated in patients with recurrent pregnancy loss when compared with control subjects. Our findings reveal a novel mechanism establishing adequate endometrial glycogen stores for pregnancy. Disruption of this histiotrophic pathway leads to adverse pregnancy outcome.

## Introduction

Human pregnancy is marred by early failure^1–3^. Approximately 15% of clinically recognized pregnancies miscarry^4,5^. When combined with pre-clinical losses, the true incidence is closer to 50%, rendering miscarriage by far the most common complication of pregnancy^4,6^. Preparation of the endometrium for pregnancy starts with the postovulatory surge in circulating progesterone levels, which in turn inhibits estrogen-dependent proliferation of the uterine epithelium, induces secretory transformation of the uterine glands and transforms stromal cells into specialized secretory decidual cells^1^. Subsequently, the luminal epithelium expresses an evolutionarily conserved repertoire of molecules essential for stable interaction and adherence of a blastocyst, thus enabling implantation^1,5^.

Glucose utilization and metabolic fate during endometrial differentiation are not well understood. Decidualization of endometrial stromal cells *in vitro* is dependent on adequate glucose concentrations. Endometrial cells cultured in glucose concentrations below 2.5 mM exhibit lower levels of decidualization, thus providing additional convincing data that increased glucose uptake is a prerequisite for decidualization^7^. Furthermore, impaired decidual transformation of the endometrium prior to conception predisposes for subsequent pregnancy failure, including recurrent pregnancy loss (RPL), defined here as 3 or more consecutive pregnancy losses^8^.

Prior to full invasion of the trophoblast and establishment of a functioning placenta, the uterine endometrium is primarily responsible for providing the nutritional support for the embryo. Early embryo development is dependent on histiotrophic nutrition, with the trophectoderm phagocytosing first the oviductal and then the uterine secretions^9^. Further, glucose must be adequately transferred from the maternal circulation^10^. The decidua prior to the onset of placental perfusion is hypoxic (mean oxygen tension rises from <20 mm Hg at 8 weeks to >50 mm Hg at 12 weeks)^11^, the embryo is thus dependent on anaerobic glycolysis for energy supply^11^. Anaerobic glycolysis critically depends on the availability of glucose. A valuable glucose source is glycogen, which accumulates in endometrial tissue^11^. Glycogen synthesis is accomplished by an intracellular glycogen synthase^12^, which is upregulated by progesterone but inhibited by estrogens^13^. Intracellular glycogen synthesis requires cellular uptake of glucose, which could be accomplished by passive glucose carriers of the GLUT family^14^. Another possible mechanism to accumulate glucose involves the high affinity Na^+^-coupled glucose transporter SGLT1, a secondary active transporter driven by the steep electrochemical Na^+^ gradient across the cell membrane^15^. SGLT1 accomplishes cellular glucose uptake even at low extracellular glucose concentrations^15^. The electrochemical Na^+^ gradient is generated by the Na^+^/K^+^ATPase^15^. SGLT1 is primarily expressed at the apical membranes of enterocytes from the small intestine and cells from the late segments (S3) of the renal proximal tubules^15–17^. To the best of our knowledge, expression of SGLT1 in the endometrium has not yet been described.

The present study explored whether SGLT1 is expressed in endometrial epithelium and decidualizing stroma, and if it affects glycogen accumulation during pregnancy and impacts on the growth and survival of the offspring.

## Results

### SGLT1 expression peaks during the window of implantation

**Figure 1:**
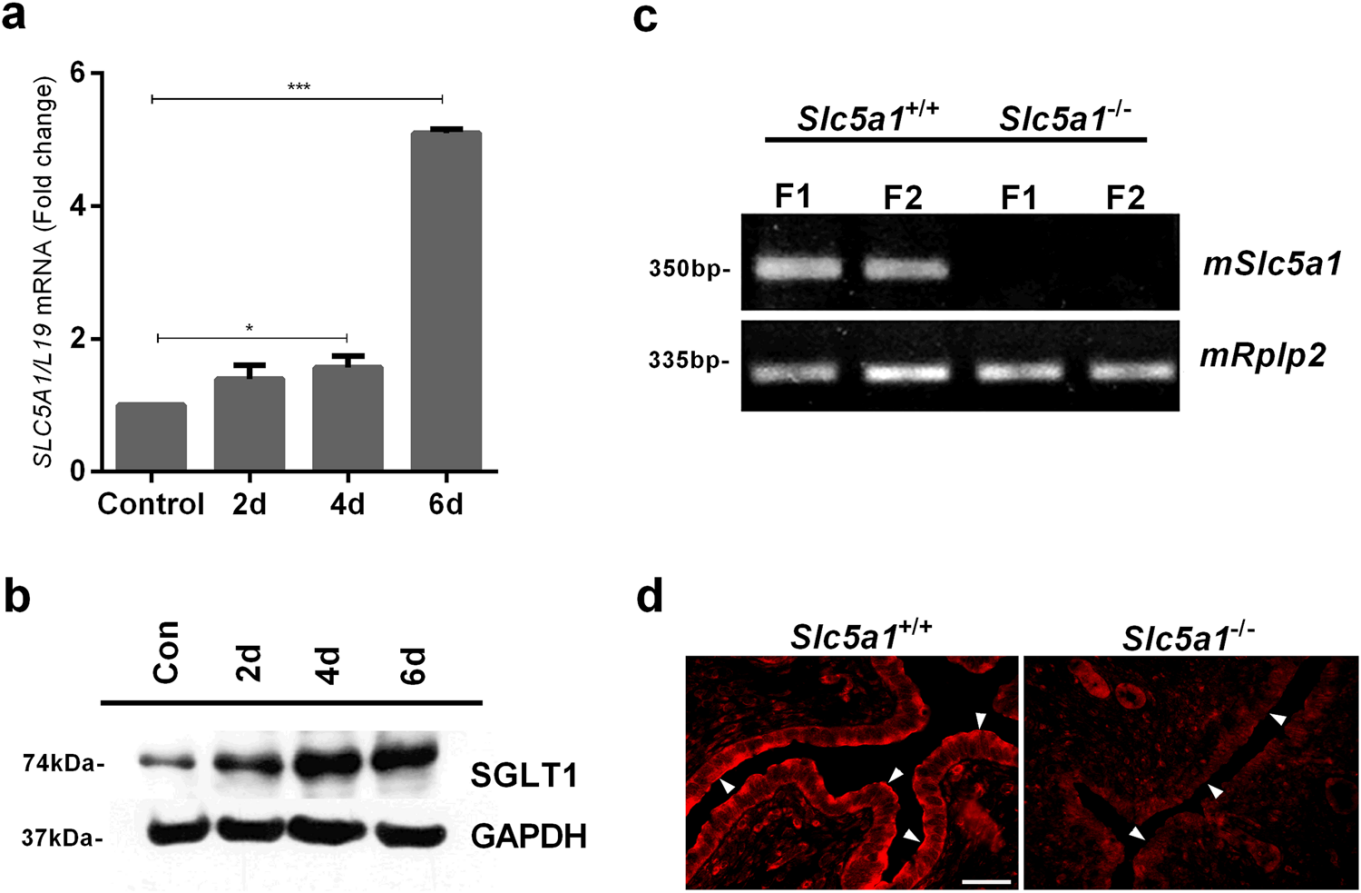
Expression of SGLT1 in human and murine endometrium. (**a**) qRT-PCR analysis of *SLC5A1 (SGLT1)* transcript levels in primary human endometrial cells (HESCs) decidualized with 0.5 M 8-br-cAMP (cAMP) and 1 μM medroxyprogesterone acetate (MPA) for 2, 4, or 6 days. Control cells remained untreated. Data were normalized to *L19* (ribosomal protein 19; housekeeping gene) and expressed as fold-change relative to transcript levels of undifferentiated (control) samples. The data are means (±SEM) of 3 independent primary cultures. *Indicates *P* < 0.05 and ***In﻿dicates *P* < 0.001 (Student’s *t*-test). (**b**) Western blot of SGLT1 in whole-cell lysates obtained from primary HESC cultures treated as indicated. GAPDH expression was used as loading control. The figures presented are cropped. Full images are in the supplementary information. (**c**) *mSlc5a1* mRNA was expressed in uteri from *Slc5a1* ^+/+^ mice while expression was absent in *Slc5a1* ^−/−^ mice. The expression of housekeeping gene *mRplp2* (*mouse ribosomal protein, large P2*) mRNA was similar in both *Slc5a1* ^+/+^ and *Slc5a1* ^−/−^ mice (n = 2). (**d**) Immunolocalization of mSglt1 protein in murine endometrium. Heterogeneous SGLT1 immunoreactivity was observed in the endometrial epithelium of *Slc5a1* ^+/+^ mice; in some regions the staining was apical (arrowheads), but most other cells exhibited variable intracellular, largely subapical staining. The staining was absent in the uterus of *Slc5a1* ^−/−^ mice. Figures shown are representative of similar findings in 3 different animals. Bar = 20 µm.

To examine if SGLT1 plays a role in endometrial glycogen accumulation, we first mined existing microarray data (GEO ID: 24461575). *SLC5A1* transcripts, encoding SGLT1, were detected in human endometrium and expression peaks during the window of implantation in the mid-luteal (mid-secretory) phase of the cycle (Supplementary Fig. 1a). Laser microdissection of human glandular epithelium coupled to RNA-sequencing showed a 10-fold increase in *SLC5A1* mRNA levels upon transition of the early- to mid-luteal phase (Supplementary Fig. 1b; Supplementary Table 1). Further, decidualization of primary human endometrial stromal cells with 8-br-cAMP and a progestin (medroxyprogesterone acetate, MPA) increased *SLC5A1* transcript and SGLT1 protein levels (Fig. 1a,b). Sglt1 was also expressed in the murine endometrium. As illustrated in Fig. 1c, *Slc5a1* transcripts were detected in uteri of wild type (*Slc5a1* ^+/+^) mice but not in Sglt1-deficient (*Slc5a1* ^−/−^) mice. Immunofluorescent staining demonstrated heterogeneous Sglt1 expression in endometrial glandular and surface epithelium of *Slc5a1* ^+/+^ animals. By contrast, Sglt1 immunoreactivity was absent in *Slc5a1* ^−/−^ mice (Fig. 1d).

### Electrogenic glucose transport across the endometrium is lost in *Slc5a1*^-/-^ mice

**Figure 2:**
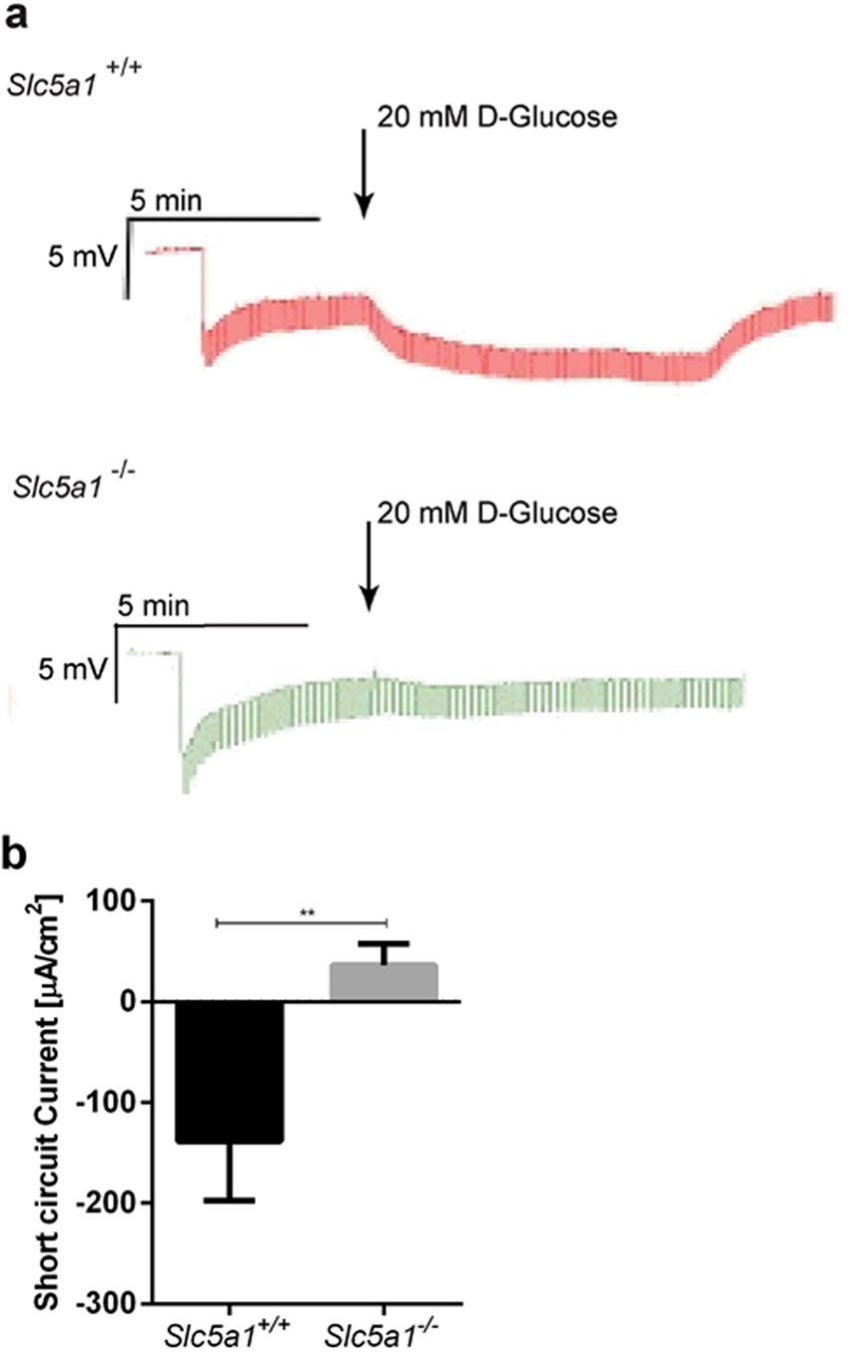
Sglt1 mediates electrogenic transepithelial glucose transport in murine uterine epithelium. (**a**) Original tracings illustrating the effect of test currents (1 µA) and of D-glucose (20 mM) on the transepithelial uterine potential difference in *Slc5a1* ^+/+^ (*upper panel*) and *Slc5a1* ^−/−^ (*lower panel*) mice. Arrows indicate the addition of D-glucose (20 mM). (**b**) Arithmetic means ± SEM (n = 3) of glucose (20 mM) induced short-circuit current across uterine epithelium from *Slc5a1* ^+/+^ *(black bar)* and *Slc5a1* ^−/−^ mice *(grey bar)*. **Indicates *P* < 0.01 (Student’s *t*-test).

To elucidate the functional significance of Sglt1, endometrial epithelium segments from *Slc5a1* ^+/+^ and *Slc5a1* ^−/−^ mice were mounted into mini-Ussing chambers. The electrogenic glucose transport was determined utilizing electrophysiological analysis. In the absence of luminal substrates, the transepithelial potential difference (Vt) amounted to 2.0 ± 0.1 mV in *Slc5a1* ^+/+^ mice and to 2.3 ± 0.1 mV in *Slc5a1* ^−/−^ mice. The transepithelial resistance (Rt) approached 6.7 ± 0.4 Ω·cm^2^ in *Slc5a1* ^+/+^ mice and 6.3 ± 1.0 Ω·cm^2^ in *Slc5a1* ^−/−^ mice. In the absence of glucose, neither the transepithelial potential difference nor the transepithelial resistance were significantly different between *Slc5a1* ^+/+^ and *Slc5a1* ^−/−^ mice. The isosmotic replacement of mannitol by glucose (20 mM) created a lumen-negative shift of the transepithelial potential difference (ΔVg), without significantly altering the transepithelial resistance. ΔVg enabled calculation of the glucose-induced current. As illustrated in Fig. 2a,b, electrogenic glucose transport across the endometrium was observed in *Slc5a1* ^+/+^ but not *Slc5a1* ^−/−^ mice. Glucose uptake into human endometrial cells was significantly decreased by the removal of extracellular Na^+^ or by addition of the SGLT1-transport inhibitor, Phlorizin (Supplementary Fig. 2).

### Sglt1 deficiency decreases endometrial glycogen content and LIF expression

**Figure 3:**
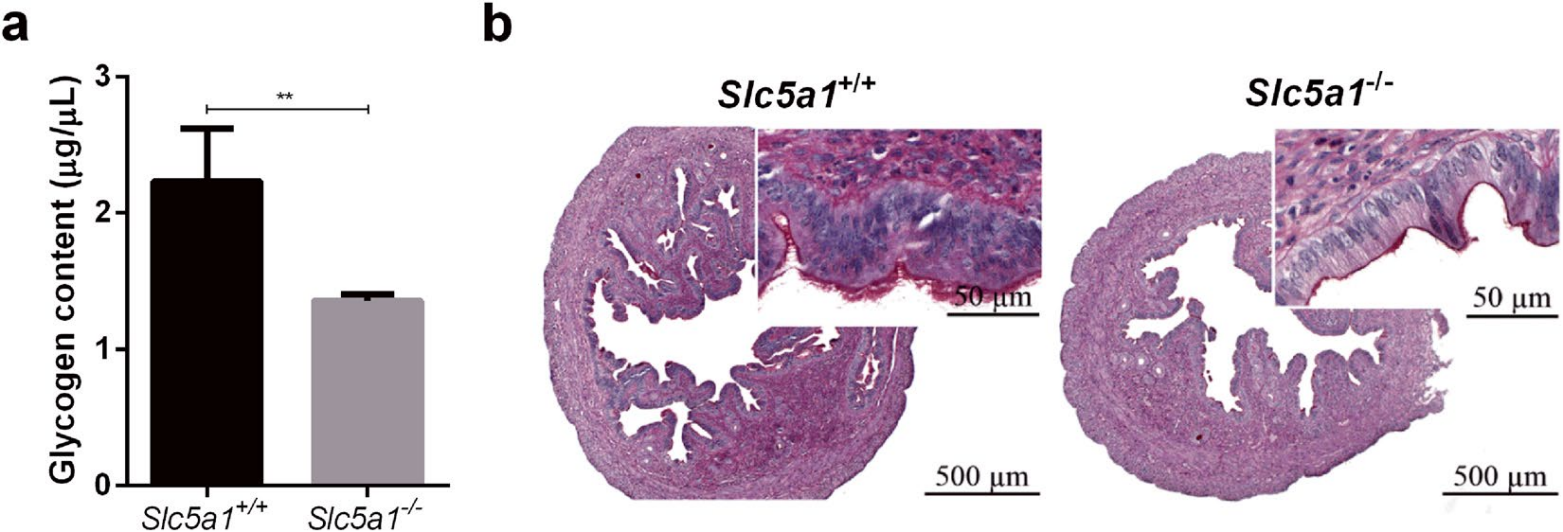
Loss of Sglt1 affects endometrial glycogen accumulation. (**a**) Glycogen abundance in the endometrium of WT and KO mice was determined, using an ELISA based method. Arithmetic means ± SEM (n = 5) of *Slc5a1* ^+/+^ mice (*black bar*) and *Slc5a1* ^−/−^ mice (*grey bar*). **Indicates *P* < 0.01 (Student’s *t*-test) (**b**) PAS staining of endometrial tissue from *Slc5a1* ^+/+^ (*left*) and *Slc5a* ^−/−^ (*right*) mice. Figures shown are representative for similar findings in the uteri from n = 5 mice. Bar = 500 µm and 50 µm.

Next, we quantified glycogen levels in the uteri of pseudo-pregnant mice (7 days post conception; d.p.c.) using a colorimetric assay^18^. The glycogen content was 61% lower in *Slc5a1* ^−/−^ mice than in *Slc5a1* ^+/+^ mice (*P* < 0.01) (Fig. 3a). Furthermore, glycogen deposits, as visualized on PAS-stained sections, were reduced in Sglt1-deficient mice (Fig. 3b). In WT mice, endometrial glycogen deposits were localized almost exclusively to glandular and luminal epithelia, supporting the conjecture that glycogen is a source of glucose for uterine histiotrophic nutrition. Notably, although the number of glands in cross-sections of the uterine wall were reduced in Sglt1-deficient mice (Supplementary Fig. 3), the expression of several key implantation genes (*Cox2*, *Wnt4*, *Bmp2*, *Hbegf*, and *Hoxa10*) was unimpaired (Supplementary Fig. 4)^19^. LIF (Leukemia inhibitory factor), which is expressed specifically by the uterine glands in response to the nidatory surge of estrogen from the ovary in mice^20^, was, however, lower in *Slc5a1* ^−/−^ than in *Slc5a1* ^+/+^ mice (Supplementary Fig. 4).

### Loss of Sglt1 is associated with reduced litter size and lower birth weight

**Figure 4:**
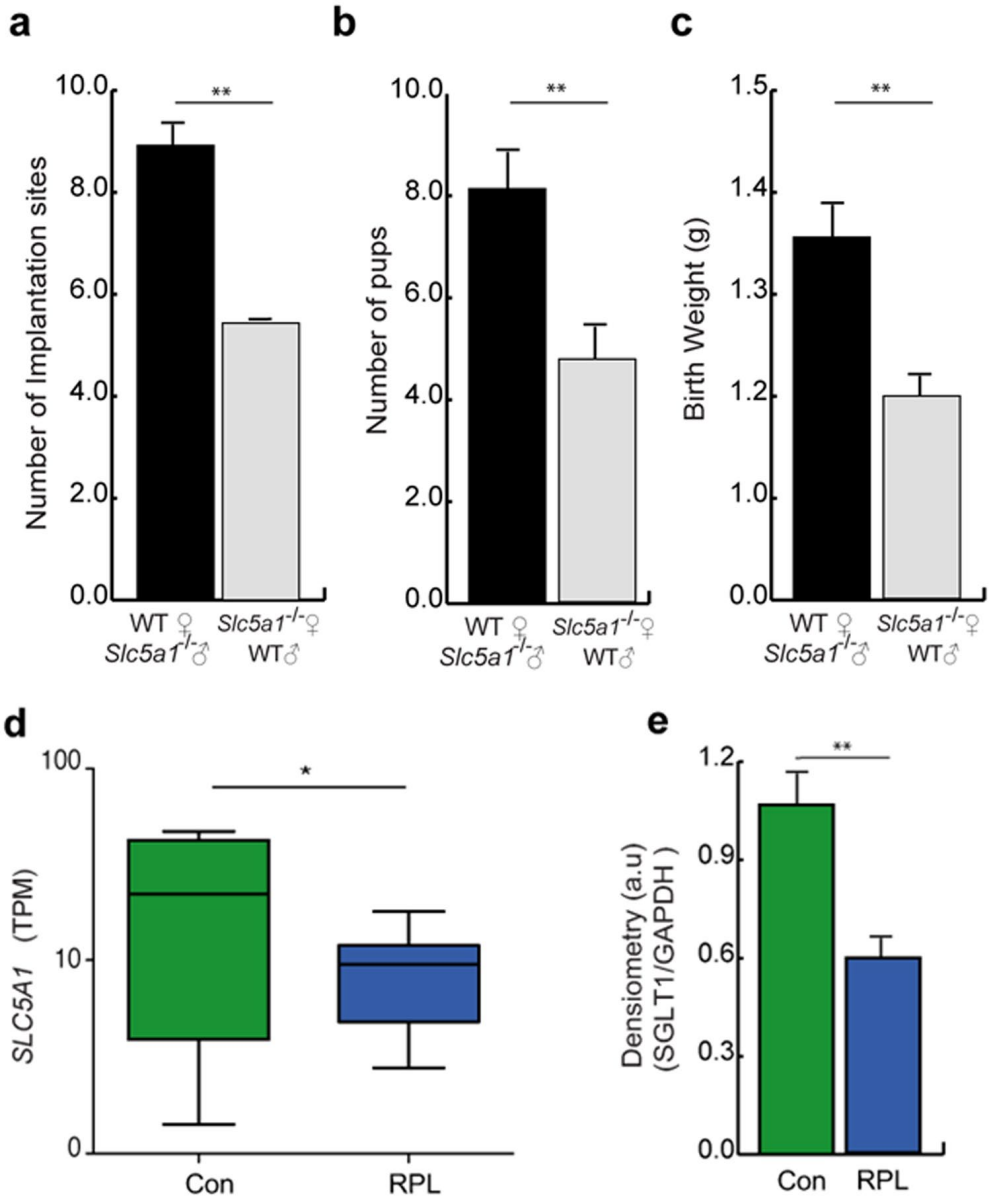
Lack of endometrial SGLT1 is associated with smaller liter size and lower birth weight in mice and recurrent pregnancy loss in humans. WT male and female mice were crossed with *Slc5a1* ^−/−^ females (*n* = 5), and males, respectively (*n* = 6). Arithmetic means ± SEM are shown. (**a**) Number of implantation sites at 7.5 d.p.c. (**b**) Pups per litter, and (**c**) Birth-weight of corresponding heterozygotic pups. (**d**) Comparison of endometrial *SLC5A1* transcripts in patients without a history of pregnancy loss (Con; n = 9) or patients with recurrent pregnancy loss (RPL; n = 9). Transcript levels are expressed as transcripts per million (TPM). The data were derived from *in silico* analysis of publicly available microarray data [Gene Expression Omnibus (GEO) Profiles; ID: GSE65102]. (**e**) SGLT1 protein levels in mid-luteal endometrial biopsies from Control subjects (n = 5) and RPL patients (n = 5) was assessed by Western blot analysis and normalized to GAPDH expression. The original Western blot data are shown in Figure S6. The densitometry data represent arithmetic means ± SEM. **Indicates *P* < 0.01 (Student’s *t*-test).

Mobilization of glycogen to glucose from the endometrium may, in part, determine embryo survival post-implantation and thus the litter size at birth. To test this hypothesis, we first determined the number of implantation sites and pups per litter in *Slc5a1* ^−/−^ female mice crossed with *Slc5a1* ^+/+^ males and in *Slc5a1* ^+/+^ females crossed with *Slc5a1* ^−/−^ males, thereby negating the potential contribution of the offspring’s genotype. The number of implantation sites at 7.5 d.p.c. was significantly lower in *Slc5a1* ^−/−^ than in *Slc5a1* ^+/+^ female mice (Fig. 4a), resulting in a lower average litter size (Fig. 4b). Moreover, the surviving pups had significantly lower birth weights, indicative of intrauterine growth restriction (Fig. 4c). To discount an effect of long term low glucose consumption on fertility, *Slc5a1* ^+/+^ mice were given a low glucose diet (LGD) and breeding studies were performed in parallel. The number of pups born to *Slc5a1* ^+/+^ females on a LGD was not significantly different from the number of pups born to *Slc5a1* ^+/+^ females on a control diet. Furthermore, the litter size of *Slc5a1* ^+/+^ females on a LGD was significantly larger than the litter size of *Slc5a1* ^−/−^ females on the same diet (Supplementary Fig. 5). To assess the relevance of endometrial SGLT1 expression for human pregnancy, mid-luteal endometrial biopsies from patients with and without a history of recurrent pregnancy loss (RPL) were analysed. As shown in Fig. 4d, endometrial *SLC5A1* transcript levels were significantly lower in timed mid-luteal biopsies from RPL patients when compared to control subjects^21^. The control group consisted of patients with infertility attributed to various causes (Supplementary Table 1). The difference in endometrial SGLT1 protein expression between the clinical groups was confirmed by Western blot analysis (Supplementary Fig. 6 and Fig. 4e; Supplementary Table 2).

## Discussion

The present study is the first report on the expression and activity of the high affinity Na^+^-coupled glucose transporter SGLT1 in the endometrium. The carrier is driven by the steep electrochemical gradient of Na^+ 15^. At a coupling ratio of 2:1, a cell membrane potential of −60 mV, and an intracellular Na^+^ concentration of 15 mM, the carrier is able to establish a glucose gradient of 10,000 to 1, i.e. an extracellular glucose concentration ten thousand times lower than the intracellular glucose concentration. In contrast to SGLT1, GLUT1^22^ and its isoforms accomplish passive transport without generating a chemical glucose gradient across the cell membrane^23,24^. Nevertheless, glucose uptake into endometrial cells is not only accomplished by SGLT1 but may also involve glucose carriers of the GLUT family. In keeping with this notion, GLUT1 protein levels in endometrial biopsies are reportedly significantly decreased in women with idiopathic infertility^25^.

Sglt1 deficiency in mice compromised cellular glucose uptake and glycogen storage in endometrial tissues. The glycogen synthase activity is highly sensitive to the concentration of glucose-6-phosphate^26^. It is thus likely that glycogen formation is a function of cellular glucose accumulation. Moreover, alterations in maternal serum glucose concentration, cellular glucose uptake or metabolism of glucose could increase the risk of implantation failure or compromise fetal development and lead to pregnancy loss. Along these lines, lack of Sglt1 significantly decreased the litter size. There was a non-significant trend towards a lower litter size in Slc5a1^+/+^ animals maintained on LGD when compared to normal diet. However, the differences in litter size between *Slc5a1* ^+/+^ and *Slc5a1* ^−/−^ mice were still significant if both genotypes received the same diet.

SGLT1 expression and activity is induced by serum- and glucocorticoid-inducible kinase 1 (SGK1)^27^, and lack of SGK1 has been shown to cause pregnancy failure^28^. It is tempting to speculate that reduced endometrial SGK1 activity compromises pregnancy in part through down-regulation of SGLT1. Effects of insulin and glucose on fertility are of great interest due to the rise of insulin resistance and type-2 diabetes prevalence among women of reproductive age. Insulin acts through the insulin receptor and IGF-I receptor, which have been identified in the endometrial stroma^29^, activating SGK1 in insulin-sensitive tissues such as muscle and fat^30^. It is further tempting to speculate that SGLT1 activity, and thus glycogen content in the endometrial tissues, also affects fetal programming.

A growing proportion of young women are diagnosed with polycystic ovary syndrome (PCOS), a multifactorial disease, characterized by typical ovarian morphology on ultrasound scan, anovulation, hyperandrogenism, and hyperinsulinemia. These patients also have increased rates of adverse pregnancy outcome, including early pregnancy loss and late obstetrical complications, such as preeclampsia and preterm labor^31^. A number of groups have proposed that abnormal glucose metabolism in the endometrium contributes to the increased risk of adverse pregnancy outcome in this population^32^. Our observation that impaired histiotrophic nutrition in early pregnancy compromises fetal growth further supports this notion.

In conclusion, SGLT1 is expressed in the endometrium and controls glycogen accumulation essential for histiotrophic nutrition in pregnancy. Lack of Sglt1 compromises endometrial glycogen storagee, resulting in reduced litter size and low birth weight in mice. Relative SGLT1 deficiency in the human endometrium at implantation may predispose for early pregnancy failure and obstetrical complications, including fetal growth restriction. In view of the present observations particular caution is warranted in pre-conception or during pregnancy if SGLT1 inhibitors are considered for the treatment of diabetes^33^.

## Materials and Methods

### Patient selection and sample collection

The study was approved by the National Health Service National Research Ethics–Hammersmith and Queen Charlotte’s & Chelsea Research Ethics Committee (1997/5065). Subjects were recruited from the Implantation Clinic, a dedicated research clinic at University Hospitals Coventry and Warwickshire National Health Service Trust. Written informed consent was obtained from all participants in accordance with the guidelines in the Declaration of Helsinki 2000.

We confirm that all methods performed, including obtaining of consent, were performed in accordance with the relevant guidelines and regulations as approved by the ethics committee.

Samples were obtained using a Wallach Endocell sampler (Wallach) under ultrasound guidance, starting from the uterine fundus and moving downward to the internal cervical ostium. Endometrial biopsies were timed between 6–10 days after the preovulatory Luteinizing Hormone (LH) surge. All biopsies were obtained in ovulatory cycles and none of the subjects were on hormonal treatments for at least 3 months prior to the procedure. The control group consisted of women with a history of conception delay due to endometriosis, male factor, tubal factor and unexplained infertility. Recurrent pregnancy loss (RPL) was defined as three or more consecutive pregnancy losses before 24 weeks gestation.

### Primary HESC cultures

Human Endometrial Stromal Cells (HESCs) were isolated from endometrial tissues as previously described^34^. Purified HESCs were expanded in maintenance medium of DMEM/F-12 containing 10% dextran-coated charcoal-treated fetal bovine serum (DCC-FBS) and 1% antibiotic-antimycotic solution. Confluent monolayers were decidualized in DMEM/F-12 containing 2% DCC-FBS with 0.5 mM 8-bromo-cAMP (8-br-cAMP; Sigma, Poole, UK) with or without 10^–6^ M medroxyprogesterone acetate (MPA; Sigma) to induce a differentiated phenotype. All experiments were carried out before the third cell passage.

### Laser microdissection coupled to RNA-sequencing

LH-timed endometrial biopsies were subjected to laser microdissection of the endometrial glands followed by RNA-sequencing. The biopsies were obtained at LH + 5 (n = 3), LH + 8 (n = 3) and LH + 11 (n = 11). The tissues were immediately frozen in liquid nitrogen and then imbedded in OCT (optimal cutting temperature) cryomatrix (VWR International, Dublin, Ireland). Ten micron slices cut in a cryotome (Leica CM-1850; Leica Microsystems Ltd, Milton Keynes, UK) were mounted on a membrane slide (MMI) and stained with 70% (v/v) cresyl violet and 30% (v/v) eosin Y. Endometrial glands were captured using the MMI CellCut laser microdissection system and isolation caps (MMI, Eching, Germany). RNA was extracted with the RNAqueous Micro Kit (Thermo Fisher, UK). RNA quality was tested by spectrophotometry (Nanodrop ND-1000) and RNA integrity was checked by Agilent 2100 Bioanalyzer system (Agilent Technologies, Santa Clara, CA, USA). For all samples, the RNA integrity number (RIN) was above 5 and the rRNA ratio [28 s/18 s] above 0.9. Amplified cDNA was obtained from total RNA using NuGen Ovation RNA-Seq System V2 (NuGen, San Carlos, CA, USA). NuGen Ovation Ultralow System V2 1–16 was applied for library preparation and sample barcoding. A 100 bp paired end run with Illumina NGS producing 150 million pair reads was performed by Wellcome Trust Centre for Human Genomics – High Throughput Genomics, Oxford, UK. Transcriptomic maps of single-end reads were generated using bowtie-2.2.3, samtools-0.1.19, and tophat-2.0.12 against the University of California Santa Cruz (UCSC) hg19 reference transcriptome (2014) from the Illumina iGenomes resource using the fr-firststrand setting. Transcript counts were assessed by HTSeq-0.6.1 using the reverse strand setting and intersection-non-empty mode and counts were assigned to gene IDs. Transcripts per million were calculated as recently described^35^. Count data from the TopHat-HTSeq pipeline were analyzed using two different methods for differential expression detection, i.e. DESeq. 2 and edgeR^36,37^.

### Animals

All animal experiments were conducted according to German law and the experiments were approved by the ethics authority (Regierung Unterfranken, Stadt Würzburg, Fachbereich Verbraucherschutz, Veterinärwesen und Lebensmittelüberwachung, Veitshöcherstr. 1a, 97080 Würzburg). The uterus was isolated from 12 week-old C57BL/6 J female WT mice (*Slc5a1* ^+/+^ ) as well as from Sglt1 deficient mice (KO, *Slc5a1* ^−/−^). Generation and breeding of *Slc5a1* ^+/+^ and *Slc5a1* ^*−/−*^ mice were described in detail previously^17^. All animals had free access to water. *Slc5a1* ^+/+^ mice were fed standard chow (C1000, Altromin Spezialfutter GmbH & Co. KG, Germany). The *Slc5a1* ^−/−^ animals received a sugar-free diet composed of 33.8% protein, 30.7% fiber and 20.5% fat as well as vitamins and minerals (C1073, Altromin). One week prior to the experiments, both WT and KO mice were fed the same sugar-free diet to exclude any diet-specific effects on function. Furthermore, to determine the effect on fertility from long term low glucose consumption, *Slc5a1* ^+/+^ mice were given a low glucose diet (LGD) for 4 weeks. Then, breeding studies were performed for a subsequent 8 weeks (on LGD) and the numbers of pups born counted.

### End-point RT-PCR

The uteri were removed and immediately submerged into the RNAlater solution (Sigma, USA). The end-point RT-PCR was performed with cDNA samples obtained from RNA preparations from two WT and two KO mice. Total cellular RNA from uterus was isolated and purified with TRIzol Plus RNA purification kit according to the manufacturer’s instructions (Ambion by Life Technologies, USA). RNA concentrations and purity were estimated spectrophotometrically by Biospec-nano (Shimadzu, Japan). The quality and integrity of RNAs were tested by agarose gel electrophoresis (data not shown). Isolated RNAs were stored at −70 °C until use. First strand cDNA synthesis was performed by High-Capacity cDNA Reverse Transcription Kit (Applied Biosystems, USA) according to manufacturer’s instructions. The synthesized cDNA was stored at −20°C. PCR was performed in a total volume of 20 µL using: 1 µL first strand cDNA (100ng), 0.4 µM specific primers, 0.2 mM dNTP mix, 1X PCR buffer and 0.025 U/μL AmpliTaq DNA polymerase (Applied Biosystems, USA) following the manufacturer’s instructions. Custom primers for *mSlc5A1* and *mRplp2* (mouse ribosomal protein, large P2; Invitrogen) were created by freely available software Primer 3.0 (*Slc5A1* F: GCCATGGACAGTAGCACCTT R: AATATCCAGCCCAGCACAAC; *Rplp2* F: TACGTCGCCTCTTACCTGCT R: AACAAGCCAAATCCCATGTC). To avoid amplification of genomic DNA, intron–spanning primers were created (*primers span* more than one *intron)*. Reaction conditions used for PCR were: initial denaturation for 3 min at 94 °C, denaturation for 30 sec at 95 °C, annealing for 30 sec at 57 °C and elongation for 45 sec at 72 °C. The non-template control (NTC) reactions (cDNA was substituted with DNase/RNase free water) were included in each PCR reaction. The PCR products were not detected in NTC reactions (data not shown). RT-PCR products were resolved by electrophoresis in 1% agarose gel stained with ethidium bromide, and visualized under UV light. The housekeeping gene *mRplp2* was used as a control for variations in the input of cDNA. The optimal number of PCR cycles within the exponential phase of the PCR reaction was 30 for both *mSlc5A1* and *mRplp2*.

### Quantitative real-time (qRT)-PCR

Total RNA was extracted from HESC cultures or uteri, using Trizol (Invitrogen, Germany). Equal amounts of total RNA (2 µg) were reverse transcribed by using the Superscript First-Strand III synthesis system for RT-PCR (Invitrogen) and the resulting cDNA used as a template in qRT-PCR analysis. The gene-specific primer pairs were designed by using the Primer Blast software.

- *SLC5A1* F: AGAGGGGAACAGACAACACA R: ACCAAAACCAGGGCATTCCA;
- *L19* F: GCGGAAGGGTACAGCCAA R: GCAGCCGGCGCAAA.
- *L19* (RPL19: Human Ribosomal Protein L19) represents a human house-keeping gene. Murine Primers:
- *Lif* F: GTCAACTGGCTCAACTCAACG R: TACGCGACCATCCGATACAGC;
- Cox2 F: CCAGCACTTCACCCATCAGTT R: ACCCAGGTCCTCGCTTATGA;
- *Wnt4* F: ACCTGGAAGTCATGGACTCG R: TCAGAGCATCCTGACCACTG;
- *Bmp2* F: GGGACCCGCTGTCTTCTAGT R: TCAACTCAAATTCGCTGAGGAC;
- *Hbegf* F: CTTGCGGCTACTTGAACACA R: GAAAGCAGGATCGAGTGAGC;
- *Hoxa10* F: GCCCCTTCCGAGAGCAGCAAAG R: AGGTGGACGCTGCGGCTAATCTCT;
- *Cyclophilin* F: TGGAGAGCACCAAGACAGACA R: TGCCGGAGTCGACAATGAT.

Cyclophilin *(Cyclo)* represents a murine house-keeping gene, and its expression was used to normalize for variances in input cDNA. Detection of gene expression was performed with KappaFast-SYBR Green (Peqlab, Germany) and qRT-PCR was performed on the BioRad iCycler iQ^TM^ Real-Time PCR Detection System (Bio-Rad Laboratories, München, Germany).

Reaction conditions used for PCR were: initial denaturation for 3 min at 95 °C, denaturation for 10 sec at 95 °C and annealing for 60 sec at 60 °C repeated for 40 cycles. Following this was the melt curve (from 60 °C–95 °C each at 5 seconds increments). The non-template control (NTC) reactions (cDNA was substituted with DNase/RNase free water) were included in each PCR reaction. The PCR products were not detected in NTC reactions (data not shown). The transcript levels of the samples were expressed as arbitrary units defined by the ΔΔC_T_ method. All measurements were performed in triplicate. Melting curve analysis and agarose gel electrophoresis confirmed amplification, specificity and (>80%) efficiency. No differences in house-keeping genes were seen between the groups.

### Western Blotting

For determination of SGLT1 protein abundance, whole cell protein extracts or biopsies were prepared by lysing in RIPA buffer. Protein yield was quantified using the Bio-Rad DC protein assay kit (Bio-Rad, Germany). Equal amounts of proteins (30 μg) were separated on 10% sodium dodecyl sulfate–polyacrylamide (SDS) gel before electrotransfer onto PVDF membrane (Amersham Biosciences, Germany). Nonspecific binding sites were blocked by overnight incubation with 5% nonfat dry milk in Tris-buffered saline with 1% Tween (TBS-T) (TBS: 130 mmol/L NaCl, 20 mmol/L Tris, pH 7.6 and 1% Tween). SGLT1 was identified by primary antibodies against human SGLT1 (1:1000, #5042, Cell Signaling, The Netherlands), and antibody against GAPDH (1:1000, #8884, Cell Signaling) served as a loading control. For detection, a secondary anti-rabbit IgG antibody conjugated with horseradish peroxidase (HRP) (1:2000, #7074, Cell Signaling) was used. Protein complexes were visualized with a chemiluminescent detection kit (WesternBright™ECL, Advansta, CA, USA). All experiments were performed in 3 or more cell cultures. Bands were quantified with ImageJ Software. Gels/blots used are in compliance with the digital image and integrity policy of this journal.

### Microscopy

The uterus was removed and immediately submerged into the fixative (4% *p*-formaldehyde) and further processed as described previously for mouse organs^38^. Immunocytochemistry was performed on 4-µm thick tissue cryosections described previously^38,39^. Following antigen retrieval and washing steps in the detergent-containing buffers, cryosections were incubated for 30 min in 1% w/v bovine serum albumin solution (in PBS) to block nonspecific binding of the antibodies, then incubated with a previously described^40^ non-commercial, affinity purified, rabbit-raised, anti-mouse Sglt1 antibody (mSglt1-Ab; 1:100 in PBS) at 4 °C overnight. The samples were then rinsed, incubated in secondary GARCY3 antibody (1.6 μg/ml in PBS;CY3-labeled goat anti-rabbit IgG; # 111-165-003, Jackson ImmunoResearch Laboratories, USA) at room temperature for one hour, rinsed, overlayed with fluorescence fading retardant Vectashield (Vector Laboratories, USA). The staining was examined under the fluorescence microscope (Opton III RS; Opton Feintechnik, Germany). The images were captured using Spot RT Slider camera and software (Diagnostic Instruments, USA) and imported and processed in Adobe Photoshop 6.0.

### Ussing chamber experiments

Experiments were performed using uterine segments from 12 week-old females KO (*Slc5a1* ^−/−^) mice and from corresponding WT mice at the estrus phase of the cycle. For the analysis of electrogenic epithelial endometrial glucose transport, Ussing chamber experiments were performed. The uterus was removed and placed in ice-cold PBS. Epithelial endometrial segments were mounted onto a custom-made mini-Ussing chamber with an opening diameter of 0.99 mm and an opening area of 0.00769 cm^2^. Under control conditions, the serosal and luminal perfusate contained (in mM) 105 NaCl, 2 KCl, 1 MgCl_2_, 1.25 CaCl_2_, 0.4 KH_2_PO_4_, 1.6 K_2_HPO_4_, 5 Na^+^ Pyruvate, 25 NaHCO_3_, and 20 Mannitol. Where indicated, (20 mM) D-glucose was added to the luminal perfusate at the expense of (20 mM) Mannitol as previously described^41^. The high glucose concentration was chosen to saturate the carrier and obtain the maximal current even in case of unstirred layers. All solutions were gassed with 95% O_2_-5% CO_2_ for at least 60 min until use in the experiment. The solutions, flow lines and perfusion chamber were maintained at 37 °C by a thermostatically controlled heating system. Reagents were purchased from Sigma (Schnelldorf, Germany) or Roth (Karlsruhe, Germany). In all Ussing chamber experiments the transepithelial potential difference (Vt) was determined continuously and the transepithelial resistance (Rt) was estimated from the voltage deflections (ΔVt) elicited by imposing test currents (I_t_). The resulting Rt was calculated according to Ohm’s law^42^. The glucose induced current was calculated from the transepithelial resistance and the voltage deflection upon replacement of mannitol with glucose. Intestine was used as a positive control.

### PAS staining and H&E

The pseudo-pregnant uteri (contains no conceptus/implantation sites; 7dpc) were collected and fixed in 4% formalin and paraffin embedded. For histology 3-5 µm thick sections were cut and stained with haematoxylin and eosin (H&E). The PAS stain allows the detection of polysaccharides such as glycogen. Tissue sections were deparaffinized and hydrated. Sections were then immersed in periodic acid solution (#11415, Morphisto, Frankfurt am Main, Germany) for 12 minutes at room temperature (RT), and then rinsed twice in distilled water. Slides were then immersed in Schiff’s reagent (#1.09033, Merck, Darmstadt, Germany) for 15 minutes at RT, followed by 10 min in running tap water. Finally, a counterstain with Hematoxylin solution was performed (4 minute incubation in Mayers Hematoxylin, 10 minutes washing in running tap water, dehydration and section mounting). The presence of glycogen with the Schiffs’ reagent gives a purple-magenta colour and hematoxylin staining gives the nuclei a blue colour. Images were taken using a digital camera ProgRes C10 plus (Jenoptik, Jena, Germany) mounted on an Zeiss Axio Imager 1 microscope (Zeiss, Oberkochen, Germany), with the ImageAccess Enterprise 6 Software (Imagic Bildverarbeitung, Glattbrugg, Switzerland). Appropriate positive and negative controls were used to confirm the adequacy of the staining.

### Glycogen concentration in tissues

The pseudo-pregnant uteri (contains no conceptus/implantation sites; 7dpc) were harvested and the glycogen concentration was determined utilizing a Glycogen Assay Kit (#MAK016, Sigma, Germany), an enzymatic assay (ELISA) resulting in colorimetric (λex = 535 nm/λem = 587 nm) measurements according to the manufacturer’s protocol.

### Cellular glucose uptake

The fluorescent glucose analogue 2-(N-(7-nitrobenz-2-oxa-1,3-diazol-4-yl)amino)-2-deoxyglucose (2-NBD-glucose; Invitrogen, Darmstadt, Germany) was used to measure the relative uptake of glucose by flow cytometry. In each condition, cells were incubated with 2-NBD-glucose (30 μM) for 1 hour at 37 °C, subsequently washed twice in cold PBS and analyzed by flow cytometry (BD Biosciences, Heidelberg, Germany) in fluorescence channel FL1. To determine the effect of SGLT1 inhibition on glucose uptake, cells were incubated with 1 mM of the SGLT1 inhibitor, Phlorizin (1 mM; #274313; Sigma). To determine the effect of Na^+^ on total glucose uptake by SGLT1 inhibition, cells were incubated for 2 h with either HEPES or Na^+^-free Hepes. The solutions were composed of (in mM): standard Hepes: 115 NaCl, 5 KCl, 1 CaCl_2_, 1.2 MgSO_4_, 2 NaH_2_PO_4_ 10 glucose, 32.2 Hepes or sodium free Hepes: 132.8 NMDG Cl, 3 KCl, 1 CaCl_2_, 1.2 MgSO_4_, 2 KH_2_PO_4_, 32.2 Hepes, 10 mannitol, 10 glucose. All chemicals were purchased from Sigma.

### Statistical analysis

Data are provided as means ± SEM; n represents the number of biological replicates. Data were tested for significance using Kruskal-Wallis test, Mann-Whitney U test or Student’s *t*-test, as appropriate. Results with *P* < 0.05 were considered statistically significant.

## Electronic supplementary material

Supplementary Information
