# Supplementary material for: Loss of Endometrial Sodium Glucose Cotransporter SGLT1 is Detrimental to Embryo Survival and Fetal Growth in Pregnancy
Source: Sci Rep. 2017 Oct 3;7:12612. doi: 10.1038/s41598-017-11674-3 (PMC5626729; doi:10.1038/s41598-017-11674-3)

Supplementary Materials for

Loss of endometrial sodium glucose co-transporter SGLT1 is detrimental to embryo survival and fetal growth in pregnancy

Madhuri S. Salker[1,2], Yogesh Singh[1], Ni Zeng[1], Hong Chen[1], Shaqiu Zhang[1], Anja T Umbach[1], Hajar Fakhri[1], Ursula Kohlhofer[3], Leticia Quintanilla-Martinez[3], Ruban R.P. Durairaj[4], Flavio S. V. Barros[4], Pavle Vrljicak[5], Sascha Ott[5], Sara Brucker[2], Diethelm Wallwiener[2], Ivana Vrhovac Madunić[6], Davorka Breljak[6], Ivan Sabolić[6], Hermann Koepsell[7], Jan J. Brosens[4] and Florian Lang[1]

Affiliations:
Departments of [1]Physiology, Cardiology & Vascular Medicine; [2]Obstetrics, Gynecology & Reproductive Medicine; and [3] Institute of Pathology and Comprehensive Cancer Centre, Eberhard-Karls Universität Tübingen, D-72076 Germany.
[4]Division of Biomedical Sciences, Warwick Medical School, and Tommy's National Centre for Miscarriage Research, University Hospitals Coventry and Warwickshire NHS Trust, Clifford Bridge Rd, Coventry, CV2 2DX, UK; [5]Warwick Systems Biology Centre, University of Warwick, Coventry, CV2 2DX, UK.
[6]Molecular Toxicology Unit, Institute for Medical Research and Occupational Health,10000 Zagreb, Croatia.
[7]Department of Molecular Plant Physiology and Biophysics, Julius-von-Sachs-Institute, University of Würzburg, D-97082, Germany.

This PDF file includes:
Tables S1-S2
Figs. S1 to S6

Table S1: Patient demographics and characteristics (Samples used in Fig. 4d)

	Age (years)	Live Birth	Number of Losses	BMI	LH+ Day
**Control***	36.6 ± 4.32	0	0	24.6 ± 4.5	7.6 ± 0.96
**RPL**	38.2± 1.09	0.3± 0.48	4.9± 1.66	25.4± 3.08	8.1 ± 1.53

Data shown are arithmetic means ± SEM (n = 9 in each group)
RPL; Recurrent Pregnancy Loss
BMI; Body Mass Index
LH+; days after the luteinizing hormone peak
* The control group consisted of 9 women with conception delay due to endometriosis (n = 1), male factor (n = 2), tubal factor (n = 1), and unexplained infertility (n = 5).

Table S2 : Patient demographics and characteristics (Samples used in Fig. 4e)

	Age (years)	Live Birth	Number of Losses	BMI	LH+ Day
**Control***	35.2 ± 2.68	0	0	22.4 ± 2.07	9 ± 0.70
**RPL**	36.4± 3.28	0	4± 1.22	24.5± 3.10	8.2 ± 1.30

Data shown are arithmetic means ± SEM (n = 5 in each group)
RPL; Recurrent Pregnancy Loss
BMI; Body Mass Index
LH+; days after the luteinizing hormone peak
*The control group consisted of 5 women with unexplained infertility.

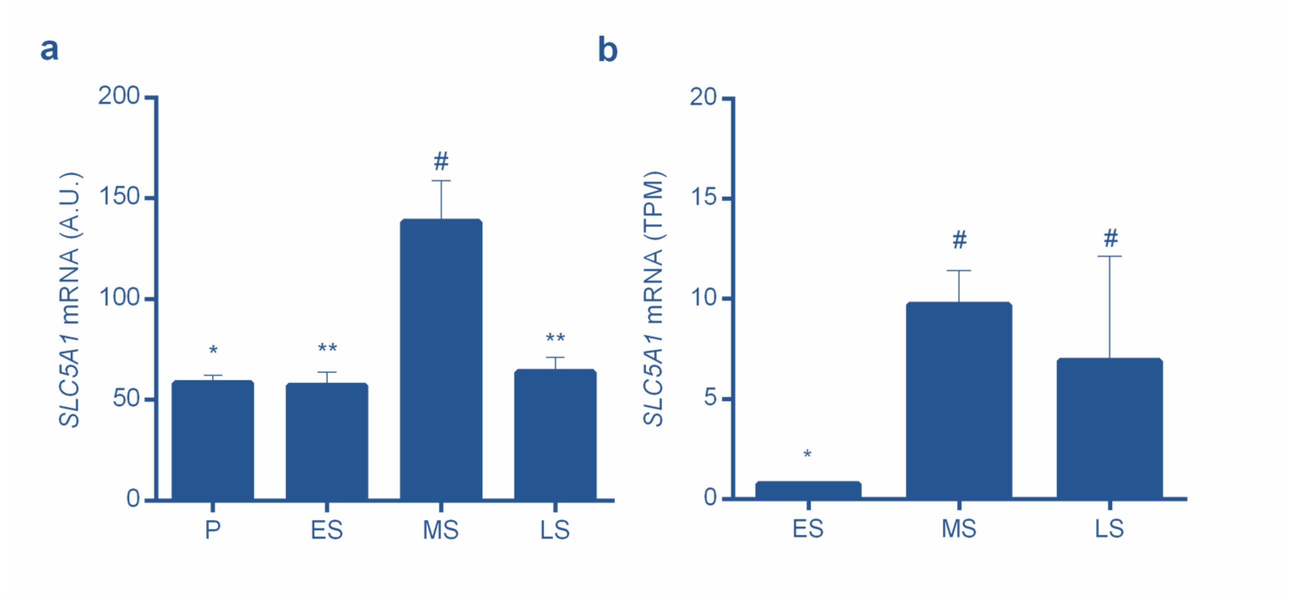

Figure S1. Expression of *SLC5A1* (SGLT1) in human endometrium.

(a) Comparison of endometrial *SLC5A1 (SGLT1)* transcripts, expressed in arbitrary units (A.U.), in proliferative (P), early (ES)-, mid (MS)-, and late (LS)-secretory endometrium. The data were derived from *in silico* analysis of publicly available microarray data [Gene Expression Omnibus (GEO) Profiles; ID: 24461575). (b) Laser microdissection of glandular epithelium coupled to RNA-sequencing showed a marked rise in *SLC5A1 (SGLT1)* mRNA, expressed in transcripts per million (TPM), upon transition of the endometrium from the non-receptive early secretory phase (ES; n=3) to the receptive mid-secretory phase (MS; n=3) and late secretory phase (LS; n=11) of the human menstrual cycle. Each bar is mean ± SEM of the data collected. Values labelled with # are significantly different from values labelled with * or ** ($P < 0.05$).

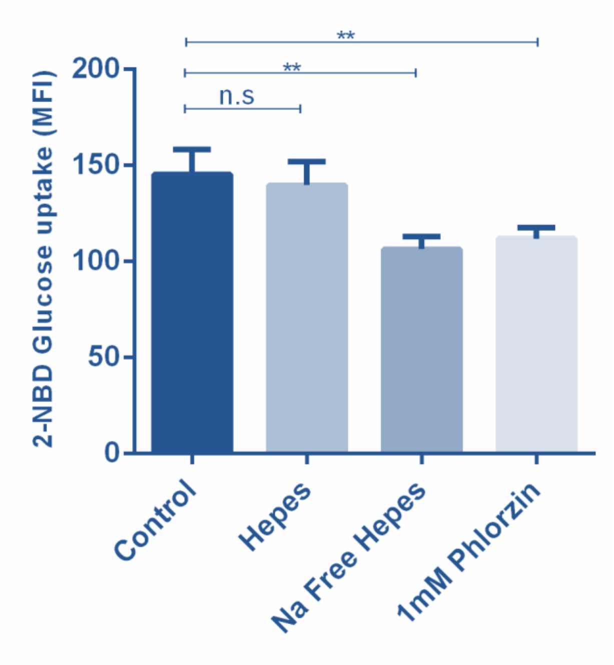

Figure S2. Cellular glucose uptake is decreased in absence of extracellular Na^{+} or in presence of SGLT1 inhibitor Phlorizin.

Arithmetic means ± SEM (n = 6) of geometric means (MFI) of the fluorescently labelled glucose analogue 2-(N-(7-nitrobenz-2-oxa-1,3-diazol-4-yl)amino)-2-deoxyglucose (2-NBD Glucose) uptake into endometrial cells in presence of Hepes, Na^{+}-free Hepes or Phlorizin (1 mM). ** indicates $P < 0.01$ (Student's *t*-test).

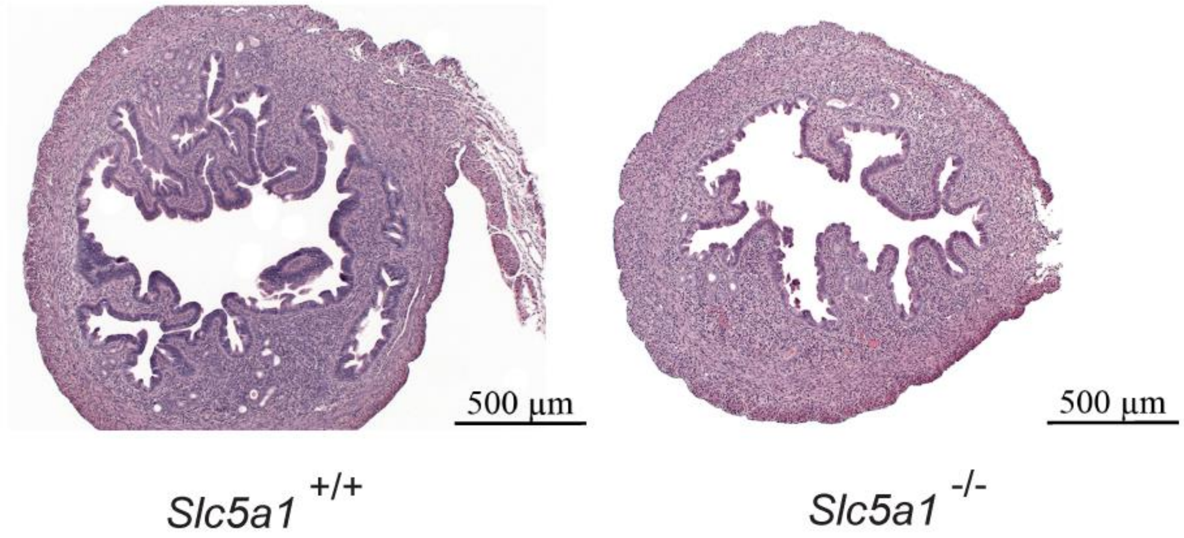

Figure S3. Loss of Sglt1 impairs glandular expansion in the mouse uterus.
Hematoxylin & Eosin (H&E) stained longitudinal uterine tissue sections were obtained from *Slc5a1*$^{+/+}$ (left) and *Slc5a1*$^{-/-}$ females (right). The abundance of endometrial glands in uterine cross-sections was reduced in *Slc5a1*$^{-/-}$ mice. Figures shown are representative for similar findings in the uteri from n=5 mice. Scale Bar 500 µm.

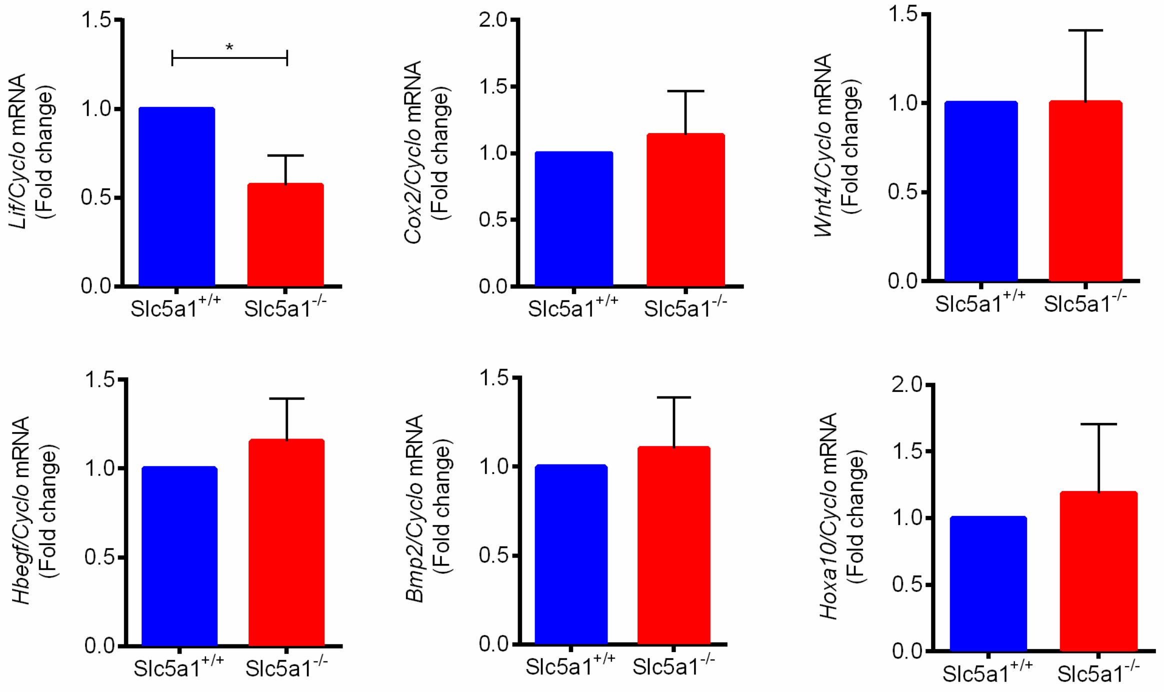

Figure S4. Expression of key uterine implantation genes in Slc5a1 (Sglt1)-deficient mice. Expression genes coding for leukemia inhibitory factor (*Lif*), cyclooxygenase-2 (*Cox2*), Wingless-Type MMTV Integration Site Family, Member 4 (*Wnt4*), Bone Morphogenetic Protein 2 (*Bmp2*), heparin-binding EGF-like growth factor (Hbegf), and homeobox A10 (*Hoxa10*) were examined by qRT-PCR in *Slc5a1*$^{+/+}$ (Blue) and *Slc5a1*$^{-/-}$ (Red) female mice at 7 d.p.c. (n = 6 in each group). Data are presented as fold change * indicates $P < 0.05$ (Student's *t*-test).

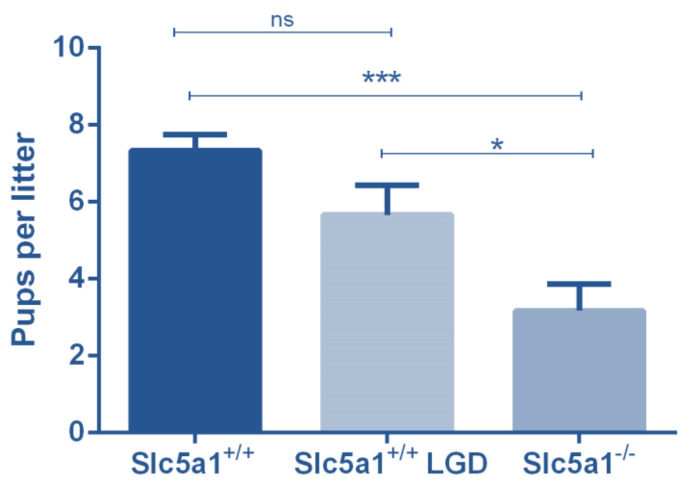

Figure S5. Impact of low glucose diet on litter size in wild type mice compared to litter size of wild type mice or *Slc5a1*$^{+/+}$ mice on regular diet.

Wild type (*Slc5a1*$^{+/+}$) mice were either given control diet or a low glucose diet (LGD) for 8 weeks. Mice were allowed to breed naturally. No significant difference was seen between *Slc5a1*$^{+/+}$ mice on either control diet or LGD. *Slc5a1* $^{-/-}$ had significantly lower number of pups. n=6 breedings, ***P<0.001 and P<0.05 when using Student's *t*-test.

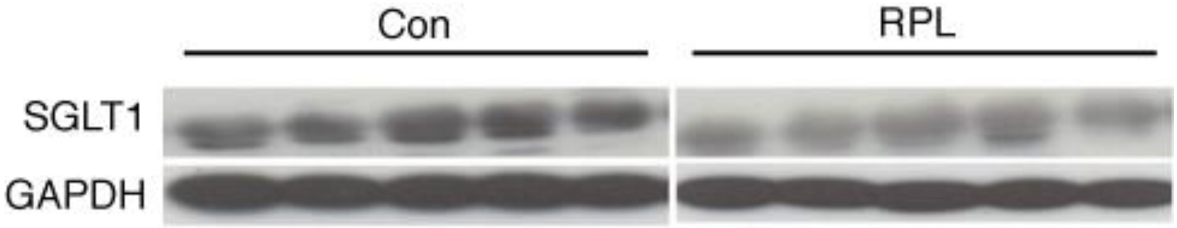

Figure S6. RPL is associated with reduced endometrial SGLT1 expression during the window of implantation.

Western blot analysis of SGLT1 expression in mid-luteal endometrial biopsies from control (n = 5) and recurrent pregnancy loss patients (RPL; n = 5).

Original Western blot shown in figure 1b.

73 kDa

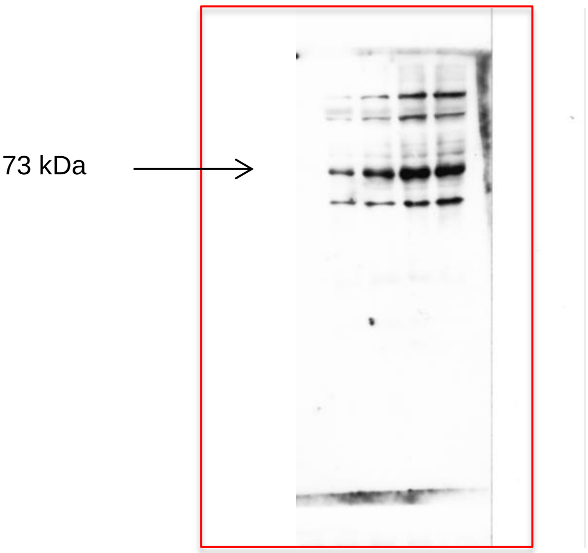

Original Western blot shown in figure 4e and figure S6.

73 kDa

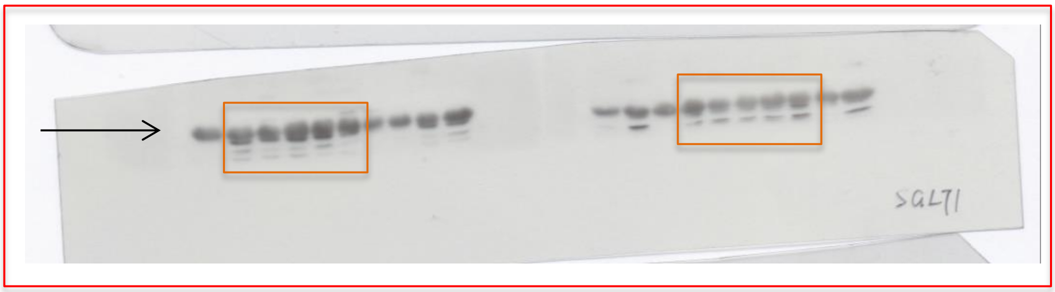

Supplement: Supplementary file 1 — Supplementary Information [file 41598_2017_11674_MOESM1_ESM.pdf]
